# Double MgO-based Perpendicular Magnetic-Tunnel-Junction Spin-valve Structure with a Top Co_2_Fe_6_B_2_ Free Layer using a Single SyAF [Co/Pt]_n_ Layer

**DOI:** 10.1038/s41598-018-20626-4

**Published:** 2018-02-01

**Authors:** Jin-Young Choi, Dong-gi Lee, Jong-Ung Baek, Jea-Gun Park

**Affiliations:** 1 MRAM Center, Department of Electronics and Computer Engineering, Hanyang University, Seoul, 133-791 Republic of Korea; 2 MRAM Center, Department of Nanoscale Semiconductor Engineering, Hanyang University, Seoul, 04763 Republic of Korea

## Abstract

A new perpendicular spin-transfer-torque magnetic-tunnel-junction (p-MTJ) spin-valve was developed to achieve a high tunneling magnetoresistance (TMR) ratio. It had a double MgO-based spin-valve structure with a top Co_2_Fe_6_B_2_ free layer and incorporated a single SyAF [Co(0.4 nm)/Pt(0.3 nm)]_3_ layer and a new buffer layer of Co(0.6)/Pt(0.3)/Co(0.4). It had a TMR ratio of 180% and anisotropy exchange field (*H*_*ex*_) of 3.44 kOe after *ex-situ* annealing of 350 °C for 30 min under a vacuum below 10^−6^ torr and a perpendicular magnetic field of 3 tesla, thereby ensuring a memory margin and avoiding read disturbance failures. Its high level of performance was due to the face-center-cubic crystallinity of the MgO tunneling barrier being significantly improved by decreasing its surface roughness (i.e., peak-to-valley length of 1.4 nm).

## Introduction

**Figure 1:**
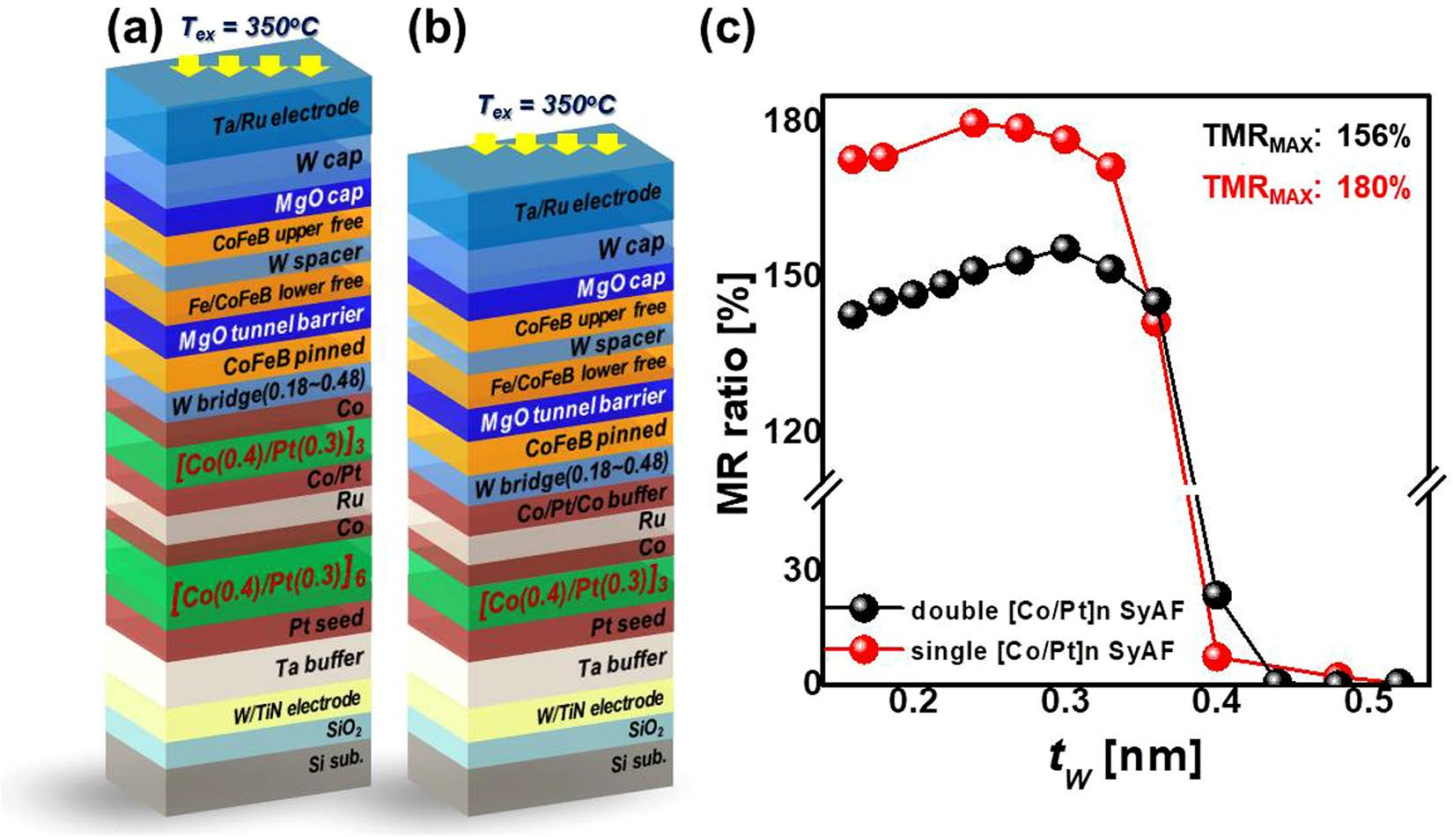
Dependency of the TMR ratio on p-MTJ spin-valve structure. Schemes of double MgO based p-MTJ spin-valve with a top Co_2_Fe_6_B_2_ free layer using (**a**) a double SyAF [Co/Pt]_n_ layers, (**b**) a single SyAF [Co/Pt]_n_ layer, (**c**) TMR ratio depending on W bridge-layer thickness (t_w_) and p-MTJ spin-valve structure.

Perpendicular spin-transfer-torque magnetic random access memory (p-STT MRAM) has been intensively researched because of its possible applications in various new memory devices and neuromorphic devices^1–4^. p-STT MRAM has many advantages over current memory devices, such as non-volatility, fast read/write speed (~10 ns), extremely low power consumption (<1 pJ/bit), high write endurance (>10^12^), and scalability^5,6^. In particular, attempts have been made to use p-STT MRAM as an embedded memory in systems-on-chip for mobile and internet-of-things applications^7,8^. In addition, terabit integration of p-STT MRAM cells has been investigated as a way to make a stand-alone memory device that would be a solution to the scaling limitations of dynamic random access memory below the 10-nm design rule. Furthermore, the p-STT MRAM concept has recently been expanded to include spin-neuron and synapse devices^3,4^. p-STT MRAM cells consist of a selective device and a perpendicular magnetic tunneling junction (p-MTJ) spin-valve^9–11^. A lot of research has gone into improving three device parameters of these spin-valves. The tunneling magnetoresistance (TMR) ratio for ensuring a memory margin should be greater than 150%. The thermal stability (*Δ* = *K*_*u*_V/*k*_*B*_T) necessary for a ten-year retention-time should be above 74, where *K*_*u*_ is the magnetic anisotropy energy, V is the volume of the free layer, *k*_*B*_ is the Boltzmann constant, and T is the temperature. The switching current density *J*_*C0*_ of about 1 MA/cm^2^ must be achieved for low power consumption. Moreover, these device parameters should be available at a back end of line (BEOL) temperature of >350 °C^12,13^. Note that BEOL process is the fabrication process to integrate the memory cells which include metal line interconnection, metal line isolation, passivation, and etc. The BEOL temperature (>350 °C) represents the temperature required during the BEOL process. To enhance these device parameters at the BEOL temperature, p-MTJ spin-valve structures have been developed to withstand temperatures greater than 350 °C. For example, the previous bottom CoFeB free layer has been changed to a top CoFeB free layer for increasing the TMR ratio^14^ and the single MgO-based p-MTJ spin-valve design has been changed to a double MgO-based p-MTJ spin-valve design for enhancing thermal stability^15–17^. In addition, it has been shown that a p-MTJ spin-valve incorporating a tungsten (W) based seed, bridging, and capping layer, instead of the tantalum (Ta) used in our previous study, enhances both the TMR ratio and thermal stability^14,18,19^. However, a double MgO-based p-MTJ spin-valve structure with a top CoFeB free layer remains a challenging target, because the roughness of the MgO tunneling barrier lower the TMR ratio^20–24^. Thus, in the study reported here, we designed a new double MgO-based p-MTJ spin-valve structure with a top CoFeB free layer using a single synthetic anti-ferromagnetic (SyAF) [Co/Pt]_n_ layer instead of a double SyAF [Co/Pt]_n_ layer that greatly reduces the roughness of the tunneling barrier, as shown in Fig. 1. First, we investigated the dependency of the TMR ratio on the body-centered-cubic (b.c.c) W bridge layer thickness for a double MgO-based p-MTJ spin-valve with a top Co_2_Fe_6_B_2_ free layer using a single SyAF [Co/Pt]_n_ layer [Fig. 1(b)] and compared it with that of a double MgO-based p-MTJ spin-valve with a top Co_2_Fe_6_B_2_ free layer using a double SyAF [Co/Pt]_n_ layer [i.e., the conventional SyAF [Co/Pt]_n_ layer: Fig. 1(a)]. Second, to understand the magnetic properties that differentiate the TMR ratio, we analyzed the static spin-torque-transfer behavior of the two different p-MTJ spin-valves by using vibrating sampling magnetometer (VSM). Third, to determine the reason for the TMR ratio difference, we observed the face-centered-cubic (f.c.c) crystallinity of the MgO tunneling barrier and Co_2_Fe_6_B_2_ pinned layer of both spin-valves by using cross-sectional high-resolution-transmission-electron-microscopy (x-HRTEM). Finally, to investigate the dependencies of the current-vs.-voltage (I-V) curve, parallel-to-antiparallel switching voltage (*V*_*P-to-AP*_), antiparallel-to-parallel switching voltage (*V*_*AP-to-P*_), reading current in the low- or high-resistance state (I_P_ or I_AP_), and the normalized TMR ratio-vs.-voltage curve on the structure of SyAF [Co/Pt]_n_ layer (i.e., a single or double), we fabricated p-MTJ spin-valve cells with a 34-nm diameter bottom electrode and 60μm-diameter top-electrode (see Supplement 1).

## Results

### TMR ratio of p-MTJ spin-valves

The dependency of the TMR ratio on the W bridge layer thickness (*t*_*W*_) was investigated as a function of the structure of SyAF [Co/Pt]_n_ layer, as shown in Fig. 1(c). For the double MgO-based p-MTJ spin-valve with a top Co_2_Fe_6_B_2_ free layer using the double SyAF [Co/Pt]_n_ layer (i.e., the conventional SyAF [Co/Pt]_n_ layer), there was a slight increase in the TMR ratio for *t*_*W*_ values up to around 0.3 nm, since the ferro-coupling strength slightly increased with *t*_*W*_. The TMR ratio abruptly decreased when *t*_*W*_ exceeded 0.36 nm, because the ferro-coupling strength between the upper [Co/Pt]_3_ SyAF layer and the Co_2_Fe_6_B_2_ pinned layer weakened abruptly. Thus, the maximum TMR ratio was only about 156% at a *t*_*W*_ of 0.3 nm. On the other hand, the TMR ratio of the spin-valve with a top Co_2_Fe_6_B_2_ free layer using a single SyAF [Co/Pt]_n_ layer slightly increased up to a *t*_*W*_ of 0.24 nm and then slightly decreased between *t*_*W*_ values of 0.24 nm and 0.36 nm. The TMR ratio then rapidly decreased for *t*_*W*_ values greater than 0.36 nm. Thus, the TMR ratio reached 180% at a *t*_*W*_ of 0.24 nm. The dependency of the TMR ratio on *t*_*W*_ for the p-MTJ spin-valve using the single SyAF [Co/Pt]_n_ layer was similar to that of the spin-valve using the double SyAF [Co/Pt]_n_ layer. However, the TMR ratio of the spin-valve using the single SyAF [Co/Pt]_n_ layer (i.e., 180%) was much higher than that of the one using the double SyAF [Co/Pt]_n_ layer (i.e., 156%). In particular, this means that the Co (0.6 nm)/ Pt (0.3 nm)/Co (0.4 nm) bridging buffer layer shown in Fig. 1(b) was well designed for anti-ferro-coupling the single SyAF [Co/Pt]_3_ layer to the Co_2_Fe_6_B_2_ pinned layer.

### Magnetic properties of p-MTJ spin-valves

**Figure 2:**
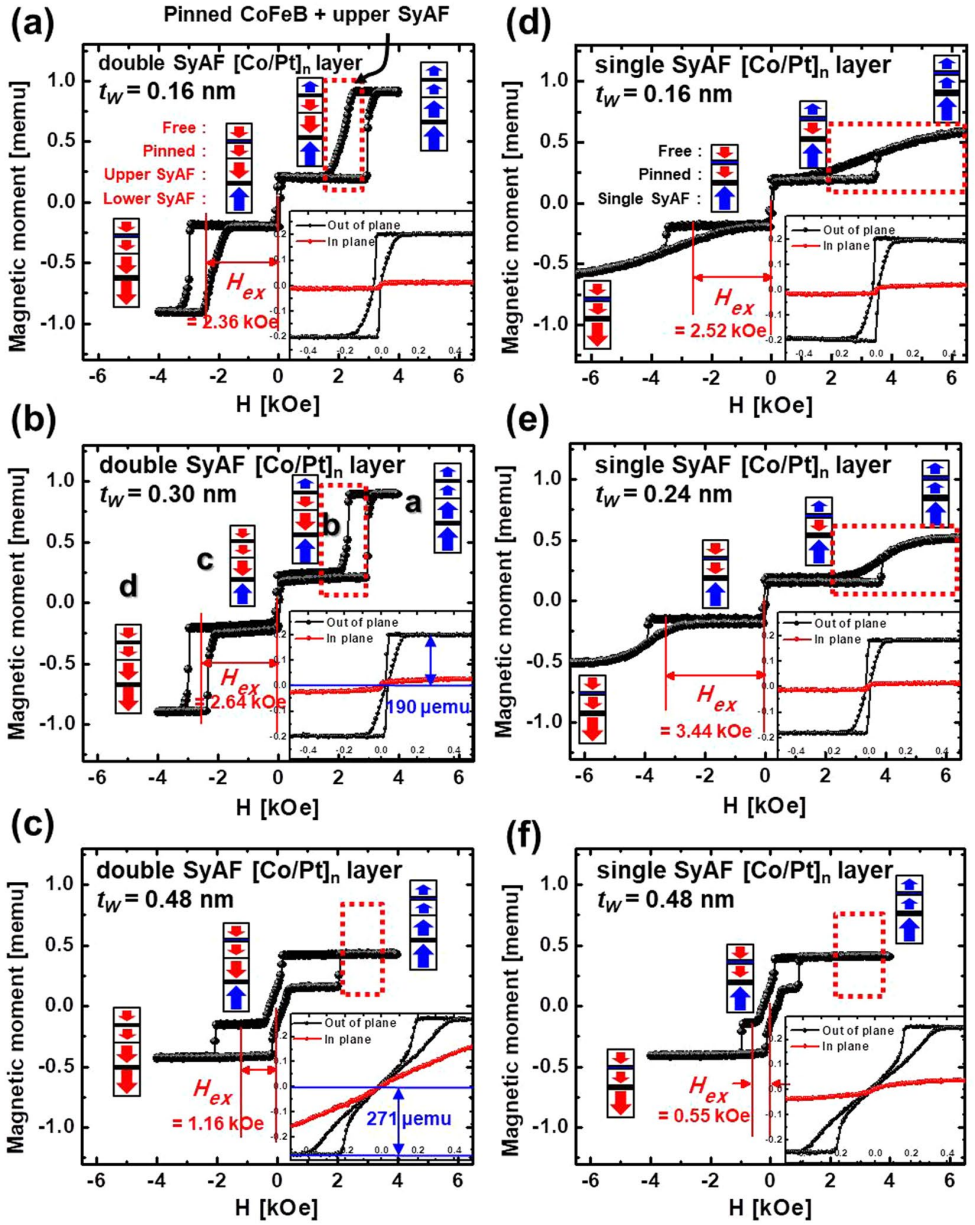
Dependency of static magnetic behavior (magnetic moments-vs.-applied magnetic field) on p-MTJ spin-valve structure and W bridge-layer thickness (t_w_). P-MTJ spin-valve with a double SyAF [Co/Pt]_n_ layer and (**a**) t_w_ = 0.16 nm, (**b**) t_w_ = 0.30 nm, (**c**) t_w_ = 0.48 nm. P-MTJ spin-valve with a single SyAF [Co/Pt]_n_ layer and (**d**) t_w_ = 0.16 nm, (**e**) t_w_ = 0.30 nm, (**f**) t_w_ = 0.48 nm.

To clarify the magnetic properties of the spin-valves with the single SyAF [Co/Pt]_n_ layers, we investigated the static *magnetic momentum-vs*.*-applied magnetic field (M-H)* loops as a function of *t*_*W*_, as shown in Fig. 2. The *M-H loops* of the spin-valves with a top Co_2_Fe_6_B_2_ free layer using the double SyAF [Co/Pt]_n_ layer [Fig. 2(a–c)] were compared with those of the spin-valves with a top Co_2_Fe_6_B_2_ free layer using the single SyAF [Co/Pt]_n_ layer [Fig. 2(d–f)]. In the case of the spin-valves using the double SyAF [Co/Pt]_n_ layer, at *t*_*W*_ of 0.16 nm, the spin-electron direction of the upper SyAF [Co/Pt]_6_ layer ferro-coupled with the Co_2_Fe_6_B_2_ pinned layer rotated from upward to downward when the external perpendicular magnetic-field (*H*) was scanned from +4 to +1.5 kOe, as shown in Fig. 2(a). Moreover, the spin-electron direction of the Co_2_Fe_6_B_2_ free layer rotated from upward to downward when *H* was scanned from + 0.2 to −0.2 kOe, showing that free layer had good perpendicular magnetic characteristics (i.e., the *M-H* loop showed good squareness) and its magnetic moment was ~0.2 memu, as shown in the inset of Fig. 2(a). The spin-electron direction of the lower SyAF [Co/Pt]_3_ layer then rotated from upward to downward and the magnetic moment of the spin-valve saturated at 0.91 memu when *H* was scanned from −1.5 to −4 kOe. As a result, at a *t*_*W*_ of 0.16 nm, the spin-valve had an anisotropy exchange field (*H*_*ex*_) of 2.36 kOe (Fig. 2(a)). Next, when *t*_*W*_ was changed from 0.16 to 0.30 nm, the squareness of the *M-H* loops of both the upper SyAF [Co/Pt]_3_ layer ferro-coupled with the Co_2_Fe_6_B_2_ pinned layer and the lower SyAF [Co/Pt]_6_ layer considerably improved (compare the red boxes in Fig. 2(a) and (b)). In addition, the squareness of the *M-H* loop of the Co_2_Fe_6_B_2_ free layer slightly improved, resulting in an increase of the TMR ratio from 143 to 156%, (see the insets of Fig. 2(a), Fig. 2(b), and Fig. 1(c)). Recall that the ferro-coupling strength between the upper SyAF [Co/Pt]_3_ layer and the Co_2_Fe_6_B_2_ pinned layer directly affect the TMR ratio; i.e., a higher ferro-coupling strength leads to a higher TMR ratio^25^. Furthermore, *H*_*ex*_ of the spin-valve increased slightly from 2.36 to 2.64 kOe, which would make the spin-valve more susceptible to read disturbance. Otherwise, when *t*_*W*_ was increased from 0.30 to 0.48 nm, the *M-H* loop of the spin-valve changed abnormally because the thicker *t*_*W*_ could not perfectly ferro-couple the upper SyAF [Co/Pt]_3_ layer with the Co_2_Fe_6_B_2_ pinned layer (see the red box in Fig. 2(c)). In particular, the squareness of the *M-H* loop of the Co_2_Fe_6_B_2_ free layer drastically deteriorated, resulting in the TMR ratio falling from 156 to 0% (the insets of Fig. 2(b) and (c)). Note that the magnetic moment increased from 190 to 271 μemu, indicating that the upper SyAF [Co/Pt]_3_ layer did not completely ferro-couple with the Co_2_Fe_6_B_2_ pinned layer since the magnetic moment of the Co_2_Fe_6_B_2_ free layer was only 190 μemu. In addition, *H*_*ex*_ of the spin-valve greatly decreased from 2.64 to 1.16 kOe, which would cause a read failure.

In contrast, for the p-MTJ spin-valve using the single SyAF [Co/Pt]_n_ layer, at a *t*_*W*_ of 0.16 nm, its *M-H* loop was completely different from that of the spin-valve using the double SyAF [Co/Pt]_n_ layer; i.e., there was no the squareness in the *M-H* loop for the single SyAF layer (see the red boxes in Fig. 2(a) and (d)). The spin-electron direction of the Co_2_Fe_6_B_2_ pinned layer gradually rotated from upward to downward and saturated as the external perpendicular magnetic-field (*H*) was scanned from +6.5 to +1.5 kOe, (Fig. 2(d)). Then, the spin-electron direction of the Co_2_Fe_6_B_2_ free layer rotated from upward to downward when *H* was scanned from + 0.2 to −0.2 kOe, meaning that the free layer showed good perpendicular magnetic characteristics and the TMR ratio was 173%. Finally, the spin-electron direction of the single SyAF [Co/Pt]_3_ layer rotated from upward to downward when *H* was scanned from −0.2 to −6.5 kOe, resulting in an *H*_*ex*_ of 2.52 kOe, which would be sufficient to avoid a read disturbance failure (Fig. 2(d)). In addition, when *t*_*W*_ was changed from 0.16 to 0.30 nm, the squareness of the *M-H* loop of the Co_2_Fe_6_B_2_ free layer slightly improved, resulting in an increase in the TMR ratio from 173 to 180% (compare the insets of Fig. 2(d), Fig. 2(e), and Fig. 1(c)). *H*_*ex*_ of the p-MTJ spin-valve considerably increased from 2.52 to 3.44 kOe, probably improving the read disturbance of p-MTJ spin-valves. Furthermore, when *t*_*W*_ changed from 0.30 to 0.48 nm, the *M-H* loop abnormally deteriorated because the thicker *t*_*W*_ could not perfectly anti-ferro-couple the single SyAF [Co/Pt]_3_ layer with the Co_2_Fe_6_B_2_ pinned layer (Fig. 2(f)). In particular, the squareness of the *M-H* loop of the Co_2_Fe_6_B_2_ free layer greatly deteriorated, resulting the TMR ratio falling from 180 to ~0% (see the insets of Fig. 2(e) and (f)). In addition, *H*_*ex*_ abruptly dropped from 2.64 to 1.16 kOe, which would probably cause a read disturbance failure. In summary, the *M-H* loop of the p-MTJ spin-valves using a single SyAF [Co/Pt]_n_ layer had an *H*_*ex*_ that was little higher than that of those using a double SyAF [Co/Pt]_n_ layer, although the total thickness of the SyAF [Co/Pt]_n_ layer was reduced considerably from 8.65 to 3.55 nm, which would not cause a read disturbance failure. However, the comparison of the *M-H* loops did not explain why the spin-valve using a single SyAF [Co/Pt]_n_ layer had a higher TMR ratio than those using the double SyAF [Co/Pt]_n_ layer. Thus, we investigated the face-centered-cubic (f.c.c) crystallinity of the p-MTJ spin-valves by using x-HRTEM. Here we should recall that the f.c.c crystallinity of the MgO tunneling barrier directly affects the probability of coherent tunneling of the spin electrons, determining dominantly the TMR ratio of a p-MTJ spin-valve^14,17–30^.

### Crystallinity of the MgO tunneling barrier

**Figure 3:**
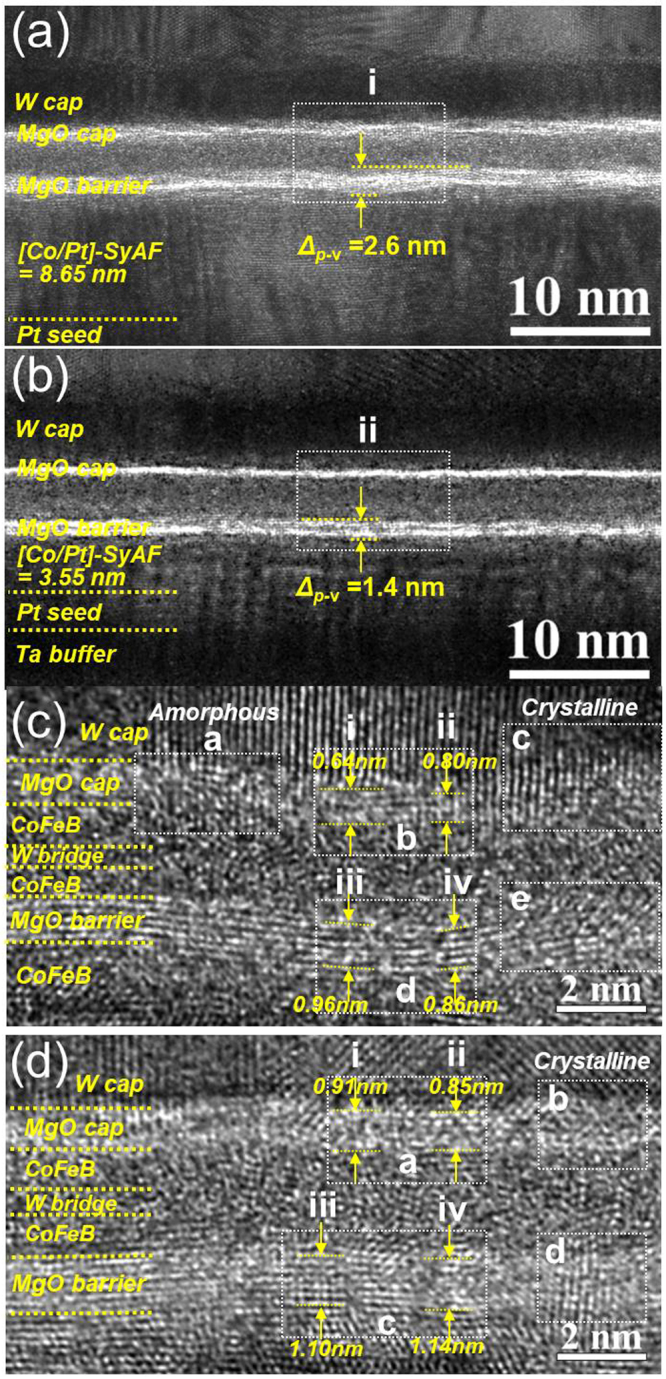
Crystallinity of MgO capping and tunneling layer depending on the p-MTJ spin-valve structure. Low magnification x-HRTEM images of p-MTJ spin-valve using (**a**) a double SyAF [Co/Pt]_n_ layer and (**b**) a single SyAF [Co/Pt]_n_ layer, (**c**) x-HRTEM images obtained from inset (i) in Fig. 3(a) and (**d**) x-HRTEM images obtained from inset (ii) in Fig. 3(b).

The f.c.c crystallinity of the MgO tunneling barrier was examined in the spin valves with the double SyAF [Co/Pt]_n_ layer at *t*_*W*_ = 0.30 nm (i.e., Fig. 2(b)) and a single SyAF [Co/Pt]_n_ layer at *t*_*W*_ = 0.24 nm (i.e., Fig. 2(e)), as shown in the x-HRTEM images of Fig. 3. The MgO tunneling barrier and capping layer of the spin-valve using the double SyAF [Co/Pt]_n_ layer had a fluctuating surface like a sinusoidal wave (Fig. 3(a) and (c)). On the other hand, the MgO tunneling barrier and capping layer of the spin-valve using the single SyAF [Co/Pt]_n_ layer had a flat surface (Fig. 3(b) and (d)). The surface roughness of the tunneling barrier and capping layer became smoother as the SyAF [Co/Pt]_n_ layer thickness was decreased from 8.65 nm (i.e., a double SyAF [Co/Pt]_n_ layer) to 3.55 nm (i.e., a single SyAF [Co/Pt]_n_ layer). In particular, the peak-to-valley of the MgO tunneling barrier (Δ_P-V_) for the single SyAF [Co/Pt]_n_ layer (i.e., 1.4 nm for t_SyAF_ = 3.55 nm) was much less than that for the double SyAF [Co/Pt]_n_ layer (i.e., 2.6 nm for t_SyAF_ = 8.65 nm), as shown in Fig. 3(a) and (b).

Since the f.c.c crystallinity of the MgO tunneling barrier directly influences the coherent tunneling of spin-electrons in the spin-valves, x-HRTEM images of regions **i** and **ii** in Fig. 3(a) and (b) were taken (Fig. 3(c) and (d)). For the spin-valve with a double SyAF [Co/Pt]_n_ layer, the fluctuating surface of the MgO tunneling barrier and capping layer (Fig. 3(c)) originated from the thicker SyAF [Co/Pt]_n_ layer (t_SyAF_ = 8.65 nm). In particular, the MgO capping layer showed almost amorphous regions (**a** and **b** in Fig. 3(c)) together with a region of locally f.c.c textured crystallinity (**c** in Fig. 3(c)) and it had a large thickness variation (i.e., ~0.64 nm at **i** and ~0.80 nm at **ii** in Fig. 3(c)). In contrast, the tunneling barrier looked almost completely f.c.c textured (**d** and **e** in Fig. 3(c)) and its thickness was ~0.96 nm at **iii** and 0.86 nm at **iv** in Fig. 3(c). Otherwise, the spin-valve with the single SyAF [Co/Pt]_n_ layer had a much smoother MgO capping layer than that using the double SyAF [Co/Pt]_n_ layer, (Fig. 3(c) and (d)) resulting in an uniform capping layer thickness (i.e., ~0.91 nm at **i** and ~0.85 nm at **ii** in Fig. 3(d)). In particular, except at the grain boundaries, the MgO capping layer was almost completely f.c.c, as shown in **a** and **b** in Fig. 3(d). Furthermore, the spin-valve with the single SyAF [Co/Pt]_n_ layer had a tunneling layer with a flatter surface than that of the one with the double SyAF [Co/Pt]_n_ layer (Fig. 3(c) and (d)). It had a uniform MgO tunneling barrier layer thickness (i.e., ~1.10 nm at **iii** and ~1.14 nm at **iv** in Fig. 3(d)). This result originated from the thickness difference between the single (t_SyAF_ = 1.40 nm) and double SyAF [Co/Pt]_n_ layer (t_SyAF_ = 8.65 nm). The MgO tunneling barrier was f.c.c. except at the grain boundaries, as shown in **c** and **d** in Fig. 3(d). In particular, the tunneling barrier (i.e., 1.10~1.14 nm) for the spin-valve using the single SyAF [Co/Pt]_n_ layer was quite thicker than that of the spin-valve with the double SyAF layer (i.e., 0.86~0.96 nm). These results indicate that the f.c.c. crystallinity of the MgO tunneling-barrier layer for the spin-valve using single SyAF [Co/Pt]_n_ layer was much superior to that of the spin-valve using the double SyAF [Co/Pt]_n_ layer. Here, we should recall that larger surface roughness in the MgO tunneling barrier reduces the hybridization of the Co-O and Fe-O atoms at the interface between the Co_2_Fe_6_B_2_ free layer and tunneling barrier which degrades the i-PMA characteristics of the p-MTJ^20,31,32^. In addition, better f.c.c crystallinity of the MgO tunneling barrier leads to a higher coherent tunneling ability of spin-electrons. Thus, both surface roughness and f.c.c crystallinity directly and dominantly affect the TMR ratio of the p-MTJ spin-valves; i.e., smaller surface roughness and better f.c.c crystallinity of the MgO tunneling barrier leads to a higher TMR ratio^14,17–30^. Thus, as revealed in the x-HRTEM image, the MgO tunneling barrier of the spin-valve using the single SyAF [Co/Pt]_n_ layer obviously showed a smaller surface roughness and better f.c.c crystallinity compared with the barrier of the spin-valve with the double SyAF [Co/Pt]_n_ layer. As a result, the p-MTJ spin-valve using the single SyAF [Co/Pt]_n_ layer (~180%) achieved a higher TMR ratio than that of the one using the double SyAF [Co/Pt]_n_ layer (~156%).

## Discussion

Double MgO-based p-MTJ spin-valve with a top Co_2_Fe_6_B_2_ free layer using a double SyAF [Co/Pt]_n_ layer (Fig. 1(a)) have been intensively studied in order to achieve TMR ratios higher than 150% in order to ensure a memory margin and enough thermal stability (*Δ*) for a ten-year retention-time at BEOL temperatures higher than 350 °C. However, the high surface roughness of the MgO tunneling barrier that originates from the thick double SyAF [Co/Pt]_n_ layer (i.e., 8.65 nm) potentially limits further enhancement of the TMR ratio. As a solution, a double MgO-based p-MTJ spin-valve structure with a top Co_2_Fe_6_B_2_ free layer using a single SyAF [Co/Pt]_n_ layer (Fig. 1(b)) was developed that has a thinner single SyAF [Co/Pt]_n_ layer (i.e., 3.55 nm) and a buffer layer bridging the single SyAF [Co/Pt]_n_ layer and the Co_2_Fe_6_B_2_ pinned layer (i.e., Co/Pt/Co layer). This device structure demonstrated a sufficient anisotropy exchange field (i.e. 3.44 kOe) for avoiding read disturbance failures and a TMR ratio about 25% higher than that of a p-MTJ spin-valve using double SyAF [Co/Pt]_n_ layer. In particular, the f.c.c crystallinity of the MgO tunneling barrier was improved by the greatly reduced surface roughness of the MgO tunneling barrier (i.e., 1.4 nm). Therefore, the double MgO-based p-MTJ spin-valve with a top Co_2_Fe_6_B_2_ free layer using a single SyAF layer would be a promising spin-valve structure for future device application such as a terra-bit integration stand-alone memory, an embedded memory, a storage class memory, etc.

## Methods

All double MgO-based p-MTJ spin-valves were fabricated using a 12-inch-wafer multi-chamber cluster-magnetron sputtering-system under a high vacuum of less than 1 × 10^−8^ torr. In particularly, the double MgO-based p-MTJ spin-valve structure with a top Co_2_Fe_6_B_2_ free layer using a double SyAF [Co/Pt]_n_ layer were fabricated as a vertical stack containing a 12-inch SiO_2_ wafer, W/TiN bottom electrode, Ta buffer layer, Pt seed layer, [Co(0.4 nm)/Pt(0.3 nm)]_6_/Co(0.6 nm) lower SyAF layer, Ru spacer layer (0.85 nm), Co(0.6 nm)/Pt(0.3 nm)/[Co(0.4 nm)/Pt(0.3 nm)]_3_ upper SyAF layers, and a Co buffer layer (0.4 nm), as shown in Fig. 1(a). The thickness of the tungsten (W) bridge layer was varied from 0.18 nm to 0.48 nm, and the p-MTJ consisted of a Co_2_Fe_6_B_2_ pinned layer (1.05 nm), MgO tunneling barrier (1.2 nm), Fe insertion layer (0.44 nm), Co_2_Fe_6_B_2_ free layer (1.0 nm), W spacer layer (0.4 nm), Co_2_Fe_6_B_2_ (1.0 nm), and MgO (1.0 nm)/W capping layer. The double MgO-based p-MTJ spin-valve with a top Co_2_Fe_6_B_2_ free layer using a single SyAF [Co/Pt]_n_ layer were fabricated wherein the ratio of the number of [Co/Pt] multi-layers between the upper and lower SyAF [Co/Pt]_n_ layer was varied from 3:6 (i.e., a double SyAF [Co/Pt]_n_ layer) to 0:3, as shown in Fig. 2 (b). In addition, the bridging buffer layer was a Co/Pt/Co layer instead of the single Co layer (compare Fig. 1(a) and (b)). Note that the bridging buffer layer is necessary to ferro-couple the Co_2_Fe_6_B_2_ free layer with the SyAF [Co/Pt]_n_ layer. The spin-valves were *ex-situ* annealed at 350 °C for 30 min under a vacuum below 10^−6^ torr and a perpendicular magnetic field of 3 tesla. The TMR ratios of the 12-inch wafer p-MTJ spin-valves were estimated by using current in-plane tunneling (CIPT) at room temperature. Afterward, the wafers were cut into 1 × 1 cm^2^ pieces. The magnetic properties (out-of-plane and in-plane) for the two different spin-valves were characterized by using vibrating sampling magnetometer (VSM) at room temperature. The crystallinity was estimated by using x-HRTEM at an acceleration voltage of 200 keV.

## Electronic supplementary material

Supplementary Information
