# Author Correction: Double MgO-based Perpendicular Magnetic-Tunnel-Junction Spin-valve Structure with a Top Co_2_Fe_6_B_2_ Free Layer using a Single SyAF [Co/Pt]_n_ Layer

**DOI:** 10.1038/s41598-018-25255-5

**Published:** 2018-04-27

**Authors:** Jin-Young Choi, Dong-gi Lee, Jong-Ung Baek, Jea-Gun Park

**Affiliations:** 1 MRAM Center, Department of Electronics and Computer Engineering, Hanyang University, Seoul, 133-791 Republic of Korea; 2 MRAM Center, Department of Nanoscale Semiconductor Engineering, Hanyang University, Seoul, 04763 Republic of Korea

Correction to: *Scientific Reports* 10.1038/s41598-018-20626-4, published online 01 February 2018

The original version of this Article contained an error in Affiliation 2 which was incorrectly listed as ‘MRAM Center, Department of Electronics and Computer Engineering, Hanyang University, Seoul, 04763, Republic of Korea’.

The correct affiliation is listed below:

‘MRAM Center, Department of Nanoscale Semiconductor Engineering, Hanyang University, Seoul, 04763, Republic of Korea.’

In addition, the Acknowledgements section in this Article contained an error.

“This work was supported by Basic Science Research Program through the National Research Foundation of Korea (NRF) grant funded by the Korea government (MSIP) (No. 2014R1A2A1A01006474) and Brain Korea 21 PLUS Program in 2014.”

now reads:

“This work was supported by Basic Science Research Program through the National Research Foundation of Korea (NRF) grant funded by the Korea government (MSIP) (No. 2017R1A2A1A05001285) and Brain Korea 21 PLUS Program in 2014.”

These errors have now been corrected in the PDF and HTML versions of the Article.

