# Supplementary material for: Double MgO-based Perpendicular Magnetic-Tunnel-Junction Spin-valve Structure with a Top Co2Fe6B2 Free Layer using a Single SyAF [Co/Pt]n Layer
Source: Sci Rep. 2018 Feb 1;8:2139. doi: 10.1038/s41598-018-20626-4 (PMC5794754; doi:10.1038/s41598-018-20626-4)
Supplement: Supplementary file 1 — Supplementary Information [file 41598_2018_20626_MOESM1_ESM.pdf]

Double MgO-based Perpendicular Magnetic-Tunnel-Junction Spin-valve Structure with a Top $Co_2Fe_6B_2$ Free Layer using a Single SyAF $[Co/Pt]_n$ Layer

Jin-Young Choi[1], Dong-gi Lee[1], Jong-Ung Baek[2] & Jea-Gun Park[*1,2]

[1] *MRAM Center, Department of Electronics and Computer Engineering, Hanyang University, Seoul, 133-791, Republic of Korea.*
[2]*MRAM Center, Department of Electronics and Computer Engineering, Hanyang University, Seoul, 04763, Republic of Korea.*

*Corresponding author:
Jea-Gun Park
17 Haengdang-dong, Seongdong-gu, Seoul 04763, Republic of Korea
Tel. (+82)-2-2220-0234; fax (+82)-2-2296-1179.
E-mail address: parkjgl@hanyang.ac.kr

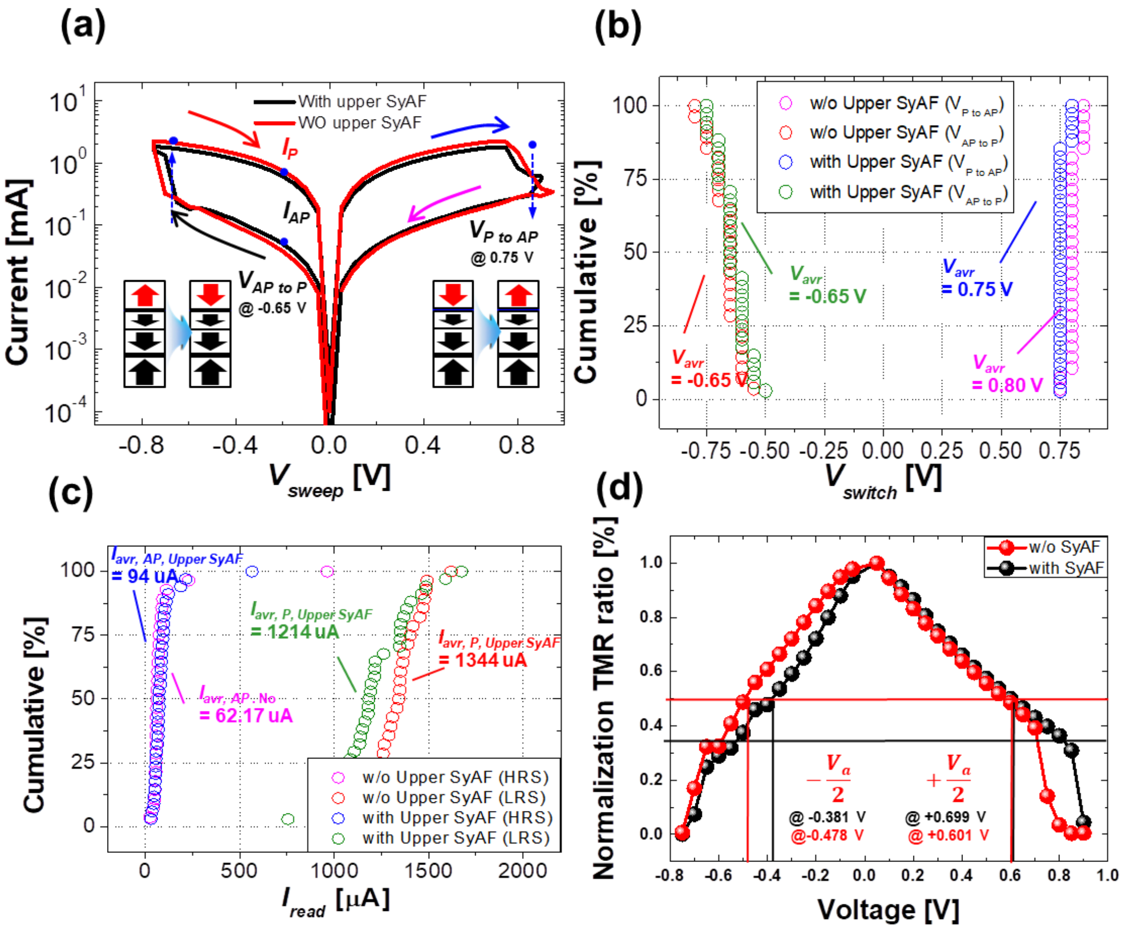

(a)
(b)
(c)
(d)
With upper SyAF
WO upper SyAF
Current [mA]
V_{sweep} [V]
I_P
I_{AP}
$V_{P\ to\ AP}$ @ 0.75 V
$V_{AP\ to\ P}$ @ -0.65 V
w/o Upper SyAF ($V_{P\ to\ AP}$)
w/o Upper SyAF ($V_{AP\ to\ P}$)
with Upper SyAF ($V_{P\ to\ AP}$)
with Upper SyAF ($V_{AP\ to\ P}$)
Cumulative [%]
V_{avr} = -0.65 V
V_{avr} = -0.65 V
V_{avr} = 0.75 V
V_{avr} = 0.80 V
V_{switch} [V]
$I_{avr,\ AP,\ Upper\ SyAF}$ = 94 uA
$I_{avr,\ P,\ Upper\ SyAF}$ = 1214 uA
$I_{avr,\ P,\ Upper\ SyAF}$ = 1344 uA
$I_{avr,\ AP,\ No}$ = 62.17 uA
w/o Upper SyAF (HRS)
w/o Upper SyAF (LRS)
with Upper SyAF (HRS)
with Upper SyAF (LRS)
I_{read} [μA]
w/o SyAF
with SyAF
Normalization TMR ratio [%]
$-\frac{V_a}{2}$
$+\frac{V_a}{2}$
@ -0.381 V
@-0.478 V
@ +0.699 V
@ +0.601 V
Voltage [V]
